# The Diet of the Asthmatic1Read before the Lake Keuka Medical and Surgical Association, August 19-20, 1902.

**Published:** 1902-12

**Authors:** George N. Jack

**Affiliations:** Depew, N. Y.


					﻿The Diet of the Asthmatic.1 J
GEORGE N. JACK, M. D., Depew, N. Y.
IT WOULD be an imprudent adventure to construct or to
attempt to construct a fixed or set diet for all or any class of
asthmatics, for such a barbarous document would be an instru-
ment of torture to persons so suffering- and a serious handicap to
i. Read before the Lake Keuka Medical and Surgical Association, August 19-20, 1902.
the physician. A properly selected individualised, progressive
and varying diet is of so great importance to the asthmatic that
on its proper selection and individualisation hinges the success of
the treatment. The diet of the asthmatic, then, becomes a daily
problem, the solution of which requires a preliminary education
in asthmatic mathematics which would constitute a thorough,
accurate and individualised knowledge of the asthmatic’s metab-
olic peculiarities together with an experienced and often
proven understanding of the action of foods under these per-
verted digestive algebraic equations.
The importance of a properly selected, varying and individu-
alised diet in the treatment of the asthmatic lies in the following
facts: (i) the blood dyscrasias producing the disease are
with but few exceptions of an alimentary digestive and blood
metabolic origin; (2) the blood is the medium through which
all absorbed foods undergo their final digestive or chemical
metabolic preparations; (3) all fat food products, after their
absorption by the lacteals, travel in all a distance of only about
twenty-seven inches, through the thoracic duct, left innominate,
superior vena cava, right heart and pulmonary arteries, direct
to the lungs for oxygenation, separation and classification before
passing through any important digestive organ for modification;
(4) all the other food products as proteids, are taken up directly
by the blood through the portal circulation, which after passing
through the liver, for modification, immediately reach the lungs
for further important, chemical metabolic preparations; (5) the
lungs, then, are not only oxygenating organs as heretofore
supposed, but they are blood, lymph, food and serum, sifting
and classifying organs as well, which permit the blood to enter
them as thick, dark, sluggish, venous blood, and to leave them
as thin, scarlet, active arterial blood, thus rendering digestion
and oxygenation co-partners.
These facts together with the determinable metabolic perver-
sions, as demonstrated by an analysis of the blood, stool, urine
and sputum, together with the individual, physical peculiarities
of the case, may be regarded as the known or given factors in
the asthmatics algebraic dietetic equation to determine the
unknown factor or the quantity and quality of food. As one
factor in the problem, the individual digestive metabolism is
of a varying, and if successful, a progressive scale, the digestive
problem necessitates a tentative answer and a frequent solution.
Among the numerous difficulties met with in prescribing a diet
for the asthmatic, the following are enumerated: (i) it is a
serious disease and one that is ever on the borderland of a
leukemia, a pernicious anemia, a cerebral hemorrhage, or a
hemorrhagic condition of the mucous membranes of the entire
respiratory tract; (2) on account of these vicious, progressive ten-
dencies a diet must as rapidly as possible be advanced to that which
will replace the waste of the disease and prevent intestinal
fermentation and putrefaction; (3) the diet must also be of such
a nature that it will not after absorption readily undergo per-
verted chemical metabolic changes in the blood; (4) a diet must in
each case eventually be established that can be persisted in for
many years; (5) the agony and torture of the disease render
all asthmatics extremists, to such an extent, that if a certain
food or diet gives them relief from their dyspnea they will
exclusively exist upon it regardless of the otherwise serious
results; (6) the misery of the disease together with its heretofore
hopeless outlook and a nervous system that is nourished with
an impoverished blood, renders the asthmatic a fit subject for a
degree of melancholy that beggars description; (7) these
depressed spirits materially interfere with the digestive function;
(8) the severity of all cases of asthma is increased by prolonged
mental depression or great anxiety, or other strenuous and not
exhilarating mental states. For obvious reasons these difficulties
are rendered much less formidable if not entirely subdued by
keeping the patient for the first few weeks at a well conducted
institute.
THE ASTHMATIC LYMPHOCYTOSIS.
The diet of these little sufferers is extremely hard to deter-
mine and regulate. However, as most of my cases have arisen
from fat breast-fed infants, whose mothers furnished them
abundant rich milk, it would indicate that overfeeding with a
readily absorbable milk, rich in lymphocytic elements, is the
most frequent cause. A few cases taken from my records sub-
stantiate this opinion, and as these cases best illustrate the
relation between diet, metabolism, blood and asthma, they will
here be quoted. I will ask you take these blood counts with a
great many “abouts,” “question marks’' and approximations,
but as they with the clinical features of the case have served to
convince me, it is hoped that they may be of practical use to
you.
v t>>	nJ	*j o C J h tJ nJ
2 h	o E 3	3	Ct	p-r- D 2	3 V	E o
g	g	f	” =■£	£ ? <u	>	B-'Sag	*S	"E
a ft ftsJS^Eo1’	2"	-’->.
«	3	S	«	E	clcgo	SE	J=S"
*	<U	Ct <t_.^.v^^w’i-^S^V	*-	fa .
(X	X c	3	E-’7-' ct rC >.7	O ?» E q	O rt
s L I 1 1 Il ^I-S 1" sh!
X	Z&S 3 W*gb3a xr.’S °	~ g J=nj££v	g> „ c
c r H rt > rt 2 >> 2 C >?	44	42	2 42	-* x rt w -S E *-
ct E 3 £	> v <t — 3 ct ~ M v	S -ti eXnJ S <u O	*c W
13 Z c	fe >- .E 2	13 ZJ	ct 4^-°	£	- co v 0	>	44 C 5
•S’Scno c'D’favJ? •- 7	c>£	q_3
^.E E fa ft o 0	ct «	P c h C .ct »- E	3 3 0
________________fa fa ~	z eg H >5 S________________5_________■£
mJ nJ	T3	nJ	rO	ct
•2 «	£ c	SfS	3.
O o	o	o £	£2?
,X	C 3	»-	n-J	J_P<	3 £
E	ct ex .	ex .	fa >,	>, o
S	u ,E g g . E S E«	•§	£
w	Ct Ct	ex fa	Ct	Ct	p	Ct "<D	Ct	Ct	JH	-E ’v
a	V V	wet	V	V	yj	V £L	V	V	4-1	tj fa
r/	33	>>o	3	3	>,	3	w	3	3	.fa	fa w
.	CX CX	nJ	O	CX	ex	nJ	fa	fa	fa	—'
>1	>>	O	fa	£	>>	O	>y	&	3	3
QQ	Z	Q	Q	£	Q	fa	Q	W_M
£» A <4	A w	E ? "
Ct,	*t	3	3	3	.J	3	3	v nJ
0	g	g	£ g 5	rt2«°
£<3	6-	3	“	°-	E	g.	" h-	g
<§ "E-S	n 3 .£ 6 °S . -H	g
<w	v	g.~3£	~
-~m *£ *- C t E	*E‘— S Er1— >-	— P E *v P	2
O	c 0 <S ~	4	“ g «O rt
_______<Z)_0 Ph M	o> co	<zj > co	O >5______________ <!
*.''<!>_________________________________1_,•_<D ,r* 1 *±1 *■* <v , *... ?
8 5 3	gii	.2	s K -^ « E u .
a O u-	g	-2^	s^-c	1? i> «J
<_•	EE'C 2-	ns C	m- 2	.8	rt - J=
f"	Ej — •S	'Uc'U	2 g 8 ►'•a n >-	-	43
w	£-=•£-	ccrt«	s^=~c •g'E'O
-	.	? s .	« o > .	.	-J3	'r>— rt -	• s 0 2
CZ	I ‘	«	—4 4^	t ‘	< 4-* 4—*	4-4	4-4	<	4—> « .	<V rT”t fV -
o^nj <u (u	<u fa E	E	ct >-	a o-S
Ct 4-»ctCCX-3	<v 7 rt> 7	7	3 ^ratnP?C:P39?
CD	E £fa£-£	^X)-E.= ct.H.3^ S
____L___________pq £ cqj5	ffl__co g pq	u)
SC33	3	3	3	3	3	33	3
a><ud)<v	<U<V<D<D<U	oj<d	<u
•sanqdomsoa	t
<U(D<D<D	<D	U	<D	<D	<D	VCD	V
cxcxjxex	exexexcxfa	exex	ex
cs m_______in m_____co n m 00_________co_______00________m
3333	33333	33	3
O <D	<D <D	<D V V <D	V	<D	<D	V
•saiXooudtuXT u u o u	<_>uuuu	u u	v
_ n	1	u	}_>_	i_	1—.	j_ >_	i-	i-.	>_	»-.
SBJE^T	<D<D<D<D	<D V V V	V	V	V	V
1	exexexex	excxexexe^	txex	ex
»n	O\	00	O	O	co	»n	O	Ch	m
3333 33333 33 3
<U	<D	<D	<D	<D	<D	<D <D	V	<D<D	V
•sajX3oqdu.iX[ ££££	£££££	t!u	£
TT'EUIQ	<D <D	V <D	<UOJ<D<D<D	OJOJ	0J
ii <>	ex ex ex ex	ex ex ex ex ex	exex	ex
co co \O -n-	O'nmmO	co \O	co
\O -f	co \O	co oi w co m	fOJ	01
, 3333 33333 3 3 3
•cnTTiirln II	0) <D	<D D	<u <D <D <D	D	OJD	<D
bJ|iqUU44	OQUQ	Q O Q Q	Q	Q	O	<D
-naujpaionu »-	*- h h !r h Jr, Jr, Jr» Jr,	Jr>
1	VO)	vv	vvvv	V	V	V	V
-oqdjouiAjoj ftftftft	ftftftftft	ftft	ft
°	2-	3 S' $ £	2, 3-	£	>c	£
8 g 8 §	- § § § 8	8	8	8	8
•saiXooonaq £	2.	£	J2 J 2.	°- A.	A. A.	o-
Os in	m C>	—,	n -	-	f	m	-	-
£ oj	rn	2?	*£	VI tZ) 2 4P If)	VI	3
.2 <n	5. SL >i ~	>.	>>	2?	2 >i	tn -	2
g^S	*	«■ -S E -g -s -S 2 -S -S -S	g
5	.2	n 3, O	<n.n°E.n	00	“	E
Q Q	"	"	2	«■	eo____________”_________«_
i	-s 6	-S -S	•£
—	i>	cone	£
o	o	o	o	o o	o
<	E	E	£	E	E
co	co	in	00	xO	O'
«	I ~	N.	M	?
<	16*--	c■	:	-	-	o	:	-
O I	. ^	,
>> u A ns ’Ti ~ ns	.ti J. _L
J 3 rt E □«	«
S^S ~	£ o 3 re	- 3 rt33 «
^fcosS	u“>'-S3Sc
SEAc -x o E	S 52 w -•£ O
o « — O - c W re <D	X S w rt
“	= = ES	c Sx-C 0> g?
*	“i^hC g-gE .	go««	egg.
“	c-o§a~	■£ ££“'§« 8 2
s	-® 8 x-° . « « 5 E 8	g =	" rt
Sa*>c-’^oC'rtr-Oo^	u2 rS	Q
s S "-2 JSS c « tfg S c
*	<v.£^Cc'Q^>'q^^	•*"	2?
= c c'j =	£2rtrS'S^w£
____________I C/)	£	>5	£
•	nS	£•
X	o	<v •
£	>	JS	c £
.	o	£	**	S'--
H	p	p 5	£ S
s	-	•=	«	o
u	5	5	5	-S3
-/	a	fa	C	C	33
a,2	o.	a.	c^
£S	>,	>>	g,«
_______________Q	Q Q o
t	sj	3
°	•£	Su
§ »	5	£ fc
£ j	sc	>
U S	O	•'JS	rv.
<2	-e	3^	«
?cn	S	=	°	3
2	rt	S-c	re
-	.tt	p	c	x
Q	o	5	rt	nS
, I _________I co___________ co	>
1— 1-. -4->	d> rr-j	.• —'GJ
CJ „, S	■—-	X	*- L_
3	3 re	S	E	rt^-o
73 > ««4	.tC	nJ	Xi
go.Eu	~	u	_33h
h	P c 1>	~	rt •’“'«□
S	C 5	S.E,.’	£ OS
”	>
rt to ••->	,—	<-»oSfS<Dx-CfC
JJuP	Mrt	«fa:fa: 3„ ere3
I	m	>	pq	pq
c	see
<U	o	o	o
'S9[tqdouiso3	£	"	“	"
o	a>	o	<u
&	CL	CL.	CL
I	CO _________JO>	00	tx_________
p	ppp
| ■spjXooqduiXf	o	o	o	o
3&IVT	o	o	o	o
CL	cl cl	cl
in	co	>n	O
P	PPP
•ssjKooqduiX]	u	a	o	u
tieiuc	3	v	3	<u
1	CL	CL	n,	Q,
'T	O	O	r*
O	\D	•’*■	O
•saiiqdojj	S	5	5	5
-naujvapnu »-	u	u
.	1 ,	u	o	<j	a»
-oqdioiuX|oj	cl	cl	cl
o	8	8°
•sajXsoonau	Q.	8	8	8.
o	O	O
O\	b*	M	00
P •	CO
CoS	-g 'S §
q o * _L_L 2
*5
a	-
E	£
<	2
•n-	co
a	i	4
W	•	w	w
<	i	o	-
II O I Z
These records and my observations indicate that bottle-fed
infants are not so susceptible to the lymphocytic asthma as the
breast-fed infants, owing presumably to a slower absorption
and assimilation. But, on the contrary, the bottle-fed infant
is more susceptible to the toxic lympho-anemic variety during
the second year and early childhood. However, if the case be
that of a fat breast-fed infant with alarming cyanotic attacks of
increasing severity, it is advisable to completely or partially
substitute the bottle for the breast.
THE ASTHMATIC TOXIC LEUKOCYTOSIS.
Under this class we have a somewhat different dietetic
problem to solve. Patients of this class usually have passed
the constructing metabolic or “growing period" and have
attained early adult life where there is a less urgent demand
upon their metabolism. The intestinal toxemia, as a rule, is well-
established, and the poisonous gases and other toxins absorbed
by the blood from this source have long interfered with the
blood’s physiologico-chemical functions. To save time and
endeavor to keep within the fifteen-minute limit, the foods that
may singly or conjointly be tried in this class according to the
condition of the blood, stool, and urine will here be men-
tioned.
These foods are: water, milk, butter, oranges, berries, pie-
plant, lemons, sour cherries—all without sugar—young onions,
celery, graham or almond bread, meats of all kinds fresh, except
lean pork, fresh fish, spinach, tomatoes, cauliflower, endives, sorel,
cucumbers, vinegar pickles, lettuce with vinegar and olive oil,
radishes, dandelions, leeks, sprouts, asparagus, cheese and eggs.
These foods in the dietetic problem may be regarded as the
ten characters or figures employed in arithmetic to express
numbers; and like these figures when used independently they
can express only the character of the material to select from,
but when combined or used according to certain principles they
serve to express all. As the student in mathematics may think
after learning the ten characters that he has mastered his sub-
ject, but the old mathematician that has devoted years to the
subject and failed on many a problem, and realises that he may
fail again, will take a different view, so here while these may be
the proper foods to use yet it is their singling out, combination
or suspension, according to the condition of the blood or
intestinal digestion, that achieves success.
The stool, blood and urine, as well as the weight, thrift and
well-being of the patient must be constantly watched. In
observing the effects of a prescribed diet we must ever bear in
mind that food, after it enters the mouth, has alimentary diges-
tion, blood digestion and metabolism to contend with before it
maketh a man. The stool is our best indicator of the alimentary
digestion, and the urine and blood, for blood digestion and
metabolism.
With all that has been said and done in regard to the chemi-
cal and microscopical analyses of feces, these analyses are of but
very little practical clinical value, for a sniff, a look, a poke,
litmus paper and a sieve, with a fresh stool will reveal more of
practical value to the experienced observer in a few minutes
time than will hours or days in a laboratory with an old stool,
a microscope and chemical reagents. The reason that this is so
is that the normal, healthy, go-lucky, hearty feeder with a
vigorous blood and a healthy active intestine will ingest food in
qualities and quantities desired, and the blood will take up
what it requires while the intestine will uncomplainingly dump
the rest. The chemical and microscopical analyses of these
normal stools would at times give us fats, starch, beef-steak,
albumoses, peptones, carbohydrates, cherry pits, and almost
anything that we were searching for, while the producer of
these stools would be jollying his time away never knowing
a sick moment with fair prospects of vigorously tiding the five-
score mark. But the things that are of practical value pertain-
ing to the stool of the asthmatic are its odor, color, consistency,
frequency, reaction and sieved out residue.
The vicious circle of nearly all varieties of asthma originate
in the intestinal canal, therefore a constant observation of the
stool is of the utmost importance in prescribing a diet. And of
all practical tests the odor with the re-action is of the most
significance. If the stool becomes acid, fermentative, with a sour
penetrating odor, it is a warning to withhold all food but water
for a time, and then to cut down or change the carbohydrates.
Then, again, if the stool becomes alkaline, with a cadaveric,
nauseating odor, it is another warning to withhold all foods but
water and to diminish the meat foods. As a rule, animal foods
are most apt to undergo deleterious changes in the intestine,
but do more readily enter into the stable and desirous com-
pounds in the blood, while, on the other hand, the vegetable
foods or carbohydrates are less apt to undergo harmful changes
in the intestine, but are more apt to undergo detrimental
changes in the blood.
If after meals the blood shows a marked leukocytosis far
exceeding the normal digestion leukocytosis, then meat must be
interdicted or diminished. But if the blood shows a pro-
nounced iodophilia, denoting an amyloid degeneration, with an
abundant outpouring of a thick, sticky, amyloid sputum, then
carbohydrates or foods rich in starch must be interdicted. In
some cases where the blood becomes decidedly amyloid, sugar
can be detected in the urine. Dr. Cott, of Buffalo, recently
sent me a case of this class. He was a thin, emaciated youth of
24 years, who had had asthma for twenty-two years. On an
analysis of the blood it was found decidedly amyloid, with 30
per cent, hemoglobin. The urine showed a specific gravity of
1030, with a trace of sugar.
THE ASTHMATIC ANEMATOSIS.
Here the main object is to feed up with a progressive diet
and to give as much fresh, bloody meat and raw eggs as the
digestion will tolerate and take care of.
CONCLUSIONS.
If on a differential count the blood shows a high percentage
of small lymphocytes milk should be diminished or withheld.
An abnormal increase in the polymorpho-nuclear neutrophilic
cells indicates an intestinal toxemia and putrefaction which
suggests the withholding of meat foods. A pronounced iodo-
philia indicates an amyloid or starchy degeneration of blood
and suggests the withholding of foods rich in starch. A thin,
watery blood with a deficient plasma and a red corpuscle poor in
hemoglobin and deficient in biomagnetism, as shown by the lack
of ruloux formation and the scattering of the red blood cor-
puscles about the field, indicates the giving of fresh bloody meat,
raw eggs and green vegetables rich in chlorophill and protoplasm.
				

